# Research Opportunities for Cancer Associated with Indoor Air Pollution from Solid-Fuel Combustion

**DOI:** 10.1289/ehp.1204962

**Published:** 2012-07-30

**Authors:** Britt C. Reid, Armen A. Ghazarian, David M. DeMarini, Amir Sapkota, Darby Jack, Qing Lan, Deborah M. Winn, Linda S. Birnbaum

**Affiliations:** 1 Division of Cancer Control and Population Sciences, National Cancer Institute, National Institutes of Health, Department of Health and Human Services (NIH, DHHS), Bethesda, Maryland, USA; 2 Integrated Systems Toxicology Division, U.S. Environmental Protection Agency, Research Triangle Park, North Carolina, USA; 3 Maryland Institute for Applied Environmental Health, University of Maryland School of Public Health, College Park, Maryland, USA; 4 Department of Environmental Health Sciences, Mailman School of Public Health, Columbia University, New York, New York, USA; 5 Division of Cancer Epidemiology and Genetics, National Cancer Institute, NIH, DHHS, Bethesda, Maryland, USA; 6 National Institute of Environmental Health Sciences, NIH, DHHS, Research Triangle Park, North Carolina, USA; 7 National Cancer Institute, NIH, DHHS, Research Triangle Park, North Carolina, USA

**Keywords:** cancer, environmental exposures, environmental health risks, epidemiology, household air pollution, indoor air pollution, public health, solid-fuel combustion

## Abstract

Background: Indoor air pollution (IAP) derived largely from the use of solid fuels for cooking and heating affects about 3 billion people worldwide, resulting in substantial adverse health outcomes, including cancer. Women and children from developing countries are the most exposed populations. A workshop was held in Arlington, Virginia, 9–11 May 2011, to better understand women’s and children’s potential health effects from IAP in developing countries. Workshop participants included international scientists, manufacturers, policy and regulatory officials, community leaders, and advocates who held extensive discussions to help identify future research needs.

Objectives: Our objective was to identify research opportunities regarding IAP and cancer, including research questions that could be incorporated into studies of interventions to reduce IAP exposure. In this commentary, we describe the state of the science in understanding IAP and its associations with cancer and suggest research opportunities for improving our understanding of the issues.

Discussion: Opportunities for research on IAP and cancer include studies of the effect of IAP on cancers other than lung cancer; studies of genetic factors that modify susceptibility; studies to determine whether the effects of IAP are mediated via germline, somatic, and/or epigenetic changes; and studies of the effects of IAP exposure via dermal and/or oral routes.

Conclusions: IAP from indoor coal use increases the risk of lung cancer. Installing chimneys can reduce risk, and some genotypes, including *GSTM1*-null, can increase risk. Additional research is needed regarding the effects of IAP on other cancers and the effects of different types of solid fuels, oral and dermal routes of IAP exposure, genetic and epigenetic mechanisms, and genetic susceptibility.

Despite considerable research on cancer and indoor air pollution (IAP) [International Agency for Research on Cancer (IARC) 2010], additional targeted research is needed in this area. In this commentary, we describe the state of the science in understanding IAP and its associations with cancer and suggest research opportunities to improve our understanding of the issues.

A workshop held in Arlington, Virginia, 9–11 May 2011, titled the “Health Burden of Indoor Air Pollution on Women and Children in Developing Countries,” was sponsored by several U.S. federal agencies and the Global Alliance for Clean Cookstoves, a public–private partnership led by the United Nations Foundation (Washington, DC). Stakeholders attending the plenary sessions included a wide array of international scientists, product developers, nongovernmental organization officials, and regulatory and policy officials, as well as community leaders and advocates from the most affected regions in Africa, India, Latin America, and China. The workshop focused on identifying research gaps concerning major health issues related to IAP. For this report we retain the use of the term “indoor air pollution” in keeping with the title of the workshop, but wish to acknowledge that the term “household air pollution” is preferred by many as more accurate in accounting for the sources of the pollution.

Exposure to IAP from the use of solid fuels is a significant public health concern affecting approximately 3 billion people worldwide and associated with an estimated 2 million deaths in the year 2000 alone (Ezzati et al. 2004; IARC 2010; World Health Organization 2009). The predominant forms of solid fuels include coal and various forms of biomass such as wood, charcoal, animal dung, and agricultural waste. Use of coal for cooking and heating is relatively uncommon globally, although it remains quite important in China and parts of central Asia (Rehfuess et al. 2006). The number of people with IAP exposure worldwide continues to increase and remains a substantial problem in many developing countries, particularly in Asia and sub-Saharan Africa. For instance, census data from India suggest that over 74% of the total population uses solid fuels for cooking (India Ministry of Home Affairs 2001). Similarly, 67% of Nigerians and 81% of Kenyans rely on solid fuels for cooking (Rehfuess et al. 2006).

## Methods

Workshop participants were divided into eight groups that addressed burns and ocular health, cancer, cardiovascular diseases, exposure assessment, infections, pregnancy/neonatology, respiratory diseases, and women’s empowerment. The authors of this report were assigned to the cancer working group. Workshop planning, execution, and post-workshop efforts were generally consistent with a previously described process to develop priorities (Montorzi et al. 2010). Efforts to identify research opportunities were strongly motivated by an announcement early in the workshop that plans were well underway by the Global Alliance for Clean Cookstoves (Washington, DC) to provide large numbers of new cook stoves and ventilation devices of various designs to affected populations through donations and other strategies over the next few years. Therefore, research that could help clarify the effectiveness of the interventions was of particular interest. To help identify research opportunities, participants also were asked to consider potential research questions in relation to the magnitude of the health burden of indoor air pollution on women and children in developing countries, the scientific unknowns, potential cost-effectiveness of the research and resulting interventions, available resources, time needed, organizational and governmental capacities, and equity concerns including the economic and political resources of those affected by IAP exposures. During breakout sessions, each group evaluated the current state of the science and identified research opportunities. The groups received input from all stakeholders in attendance during the plenary sessions.

IARC has classified IAP from coal combustion as a known human carcinogen (Group 1) and has classified IAP from the combustion of biomass fuel as a probable human carcinogen (Group 2A) (IARC 2010). IARC works with an international group of experts to comprehensively evaluate the world literature on potential carcinogens and makes qualitative assessments as to which specific agents cause cancer in humans. In October 2006, the Expert Working Group for *IARC Monograph* Vol. 95 (IARC 2010) evaluated the carcinogenicity of the household use of solid fuels and high-temperature frying. The report was updated with information on “coal only” in 2012 (IARC 2012) after the IAP meeting in 2011. We considered *IARC Monograph* Vol. 95 to be comprehensive through 2006 and limited additional searches to journal articles concerning human studies based on primary data analyses published in English after 1 January 2006. To identify articles that examined cancer associated with household use of solid fuels, we performed PubMed (National Library of Medicine, National Institutes of Health, Washington, DC) searches using the terms “indoor air pollution” OR “household air pollution” OR “solid fuel” OR “biomass” OR “wood smoke” OR “smoky coal” OR “crop residue” AND “cancer.” After eliminating duplicate manuscripts and manuscripts concerning noncancer outcomes, irrelevant exposures, or secondary analyses, we identified 14 new publications, in addition to the *IARC Monograph* published in 2010, to consider when prioritizing research opportunities. All of the publications included in the 2012 update of *IARC Monograph* Vol. 95 (IARC 2012) were identified and included in our assessment.

## IAP and Cancer: What We Know

Cancers that have been associated with IAP include cancers of the lung, upper aero-digestive tract, and cervix. Lung cancers are the most studied and well characterized of the IAP–cancer associations (IARC 2010). IARC based its determination that household exposure to coal combustion by-products causes lung cancer in humans principally on strong studies that adequately addressed tobacco use and other relevant factors as confounders, including four case–control studies from China that reported statistically significant associations between lung cancer and exposure to coal combustion (Dai et al. 1996; Lan et al. 2000; Wu-Williams et al. 1990; Xu et al. 1989). Lung cancer prevalence increased with increasing amounts of coal used (*n* = 244) (Lan et al. 2000) and years a coal stove was used for heat in the bedroom [*n* = 240 and *n* = 1,924, for Dai et al. (1996) and Wu-Williams et al. (1990), respectively].

Lung cancer associations were evident irrespective of whether coal was used for heating or cooking (Lan et al. 2000) or whether the type of coal was smoky or non-smoky (Dai et al. 1996; Wu-Williams et al. 1990). Strong evidence came from a retrospective cohort study of farmers exposed throughout their lifetimes to smoky coal (*n* = 21,232), which reported reduced risks of lung cancer following transition to use of a stove with a chimney (Lan et al. 2002). Increased adenocarcinoma of the lung was observed among users of coal or anthracite as cooking fuel in a case–control study in Taiwan (*n* = 1,332) (Lee CH et al. 2001).

Reports published since the IARC Working Group was convened in 2006 continue to fully support IAP due to coal use as a cause of lung cancer. The International Lung Cancer Case–Control Consortium members pooled data from multiple case–control studies across North America, Europe, and Asia (Hosgood et al. 2010). Based on 5,105 cases and 6,525 controls, lung cancer was increased among predominant coal users [odds ratio (OR) = 1.64; 95% confidence interval (CI): 1.49, 1.81]. This association was strong for coal users in Asia (OR = 4.93; 95% CI: 3.73, 6.52) and was evident among Asian women and men, smokers and nonsmokers, and nonsmoking women. Harbin, China, was the site of a lung cancer case–control study (*n* = 654) that, controlling for multiple potential confounding factors, found that lung cancer was associated with the use of coal for fuel as well as for cooking (Galeone et al. 2008). Further research in Xuan Wei County, including a case–control study consisting of 996 participants, has focused on subtypes of coal (Lan et al. 2008). Compared to users of non-smoky coal or wood, persons exposed to smoky coal had seven times the odds of lung cancer, a finding that was evident in both men and women. Relative risks were highest for exposure to coal from Laibin, and associations increased with an increasing concentration of benzo[*a*]pyrene in the region’s coal. Outside of China, an association between coal exposure and lung cancer was also reported by a multicenter case–control study from India (*n* = 2,579) (Sapkota et al. 2008).

Studies from several countries support previous findings and in some cases add region-specific details. A retrospective cohort study conducted in the Southwest Guizhou Autonomous Prefecture, China (*n* = 1,386), an area where coal with a high arsenic content is used for fuel, found that male and female residents with chronic arsenic poisoning were more likely than the general Chinese population to die from lung cancer (Chen et al. 2007). Wood-smoke particles produced more extensive DNA damage as measured by strand breaks and formamidopyrimidine DNA glycosylase sites than traffic-generated particulate matter in a laboratory-based study conducted on lung epithelium and monocytic cell lines (Danielsen et al. 2009). An analysis of samples collected from participants in a case–control study of Indian women (*n* = 172) suggested that chronic exposure to biomass smoke activates Akt, a protein that has been implicated in the development of a wide range of human cancers (Mondal et al. 2010).

As summarized in the 2010 *IARC Monograph*, the concentration of polycyclic aromatic hydrocarbons (PAHs) in emissions from indoor coal combustion is associated with lung cancer, and both the cytochrome P450 and aldo-keto-reductase pathways, as well as polymorphisms in DNA repair and phase II pathways, have been shown to modify the association (IARC 2010). Evidence linking PAHs in wood-smoke emissions to cancer is limited, and other components of wood-smoke IAP may also be important. At present, very little is known regarding the role of other chemical contituents of IAP in carcinogenesis—a potential area for future research.

Evidence continues to grow that interventions that reduce IAP exposures substantially reduce lung cancer mortality. In a retrospective cohort study of more than 42,000 farmers with an average follow-up of 16 years, the use of high-efficiency portable stoves was associated with 40% decrease in deaths from lung cancer in men and 60% in women when compared with traditional stoves (Hosgood et al. 2008). The authors speculate that this may have been the result of lower exposure to combustion by-products because it was no longer necessary to open the stove multiple times each day to add coal. A retrospective cohort study conducted in China (*n* = 21,232) reported a decrease in lung cancer mortality after chimneys were installed in homes with improper ventilation (Lee KM et al. 2010).

There are significantly fewer studies of lung cancer in association with IAP from the combustion of biomass, and the studies that are available do not evaluate associations according to the specific type of fuel used, making comparisons difficult. The IARC Working Group noted several strong case–control studies across several geographic regions to support their conclusion that IAP from biomass combustion is a possible carcinogen (Group 2A). A case–control study from Europe (*n* = 5,979) (Lissowska et al. 2005) and two case–control studies from Taiwan [*n* = 234 and *n* = 1,332, for Ko et al. (1997) and Lee CH et al. (2001), respectively] all reported that lung cancer was associated with the use of wood as a cooking fuel, with stronger associations with squamous cell carcinoma and adenocarcinoma than other histological subtypes of lung cancer (Ko et al. 1997; Lee CH et al. 2001). In a case–control study from Japan (*n* = 857), smoke exposure from wood or wood and straw was associated with lung cancer only among those exposed to the smoke before 30 years of age (Sobue 1990). Exposure to wood smoke for ≥ 50 years was associated with lung cancer in a case–control study from Mexico (*n* = 386) (Hernandez-Garduno et al. 2004). In a case–control study in Montreal (*n* = 2,746), use of wood for heating and cooking was associated with lung cancer (Ramanakumar et al. 2007). Finally, in a case–control study in India (*n* = 113), lung cancer was associated with the use of biomass for cooking (primarily wood, dung, and crop residues) (Behera and Balamugesh 2005). Two small cross-sectional studies from Sweden [*n* = 24 and *n* = 23, for Gustafson et al. (2007, 2008) respectively] reported that exposure to 1,3-butadiene, benzene, formaldehyde, PAHs, and acetaldehyde as measured from air samples was highly correlated with exposure to indoor wood burning for heating homes.

Since the IARC Working Group evaluated the evidence in 2006, a pooled analysis of seven case–control studies reported a modest association between lung cancer and wood-smoke exposure (Hosgood et al. 2010). In contrast, no evidence of an overall association of the use of wood for cooking and lung cancer was observed in a case–control study in India (*n* = 2,579), even with long duration of use (Sapkota et al. 2008).

Awareness of the health risks of exposure to combusted coal or biomass may be limited in many populations. For example, in a cross-sectional study in Nepal, awareness of lung cancer associations with these exposures was high among the well educated, but not among the illiterate, who may be the ones most likely to be exposed (Chawla et al. 2010).

Some associations have been reported between upper aero-digestive tract cancers and IAP. *IARC Monograph* Vol. 95 mentions studies of nasopharyngeal cancer and other upper-digestive tract cancers, but weaknesses of the studies precluded the IARC Working Group from drawing conclusions about these cancers (IARC 2010). A subsequent case–control study in India (*n* = 2,579) reported an association between the exclusive use of wood as a solid fuel and hypopharyngeal cancer but not laryngeal cancer (Sapkota et al. 2008).

Human papillomavirus (HPV) infection is a necessary cause of cervical cancer; all other factors only modify risk (Schottenfeld et al. 2006). The IARC Working Group noted one case–control study (*n* = 366), albeit with methodological limitations, that indicated an association between wood burning and cervical cancers among HPV-infected women (Velema et al. 2002). Two case–control studies have been reported since the 2006 IARC review. A study from Taiwan (*n* = 1,479) found no association, possibly because few women with cervical high-grade squamous intraepithelial lesions were also exposed to coal or wood combustion by-products (Lee CH et al. 2010). A case–control study of Colombian women (*n* = 183) reported that the risk of cervical cancer was stronger among HPV-infected women exposed to wood smoke in the kitchen for ≥ 16 years than in HPV-infected women without wood-smoke exposure (OR = 5.3; 95% CI: 1.9, 14.7) (Sierra-Torres et al. 2006).

## Priority Research Opportunities

The cancer working group identified four topics as potential high-priority opportunities for research in the near term. Studies of interventions such as chimneys and efficient cook stoves should be carried out. The opportunities described below could be conducted in the context of intervention studies to determine whether different types of solid fuel are associated with different types of cancer, whether some populations are more susceptible than others and what mechanisms need to be accounted for and to identify routes of exposure that must be accounted for to accurately quantify dose.

*Estimate effects of household solid-fuel combustion on cancers other than lung cancer.* Many carcinogens such as PAHs and metals are present in both tobacco smoke and smoke from solid-fuel combustion products (IARC 2004, 2010), which suggests that other cancers associated with tobacco smoking may also be associated with exposure to IAP. Studies cited by the IARC Working Group in 2006 were not sufficient to allow firm conclusions about effects on cancers other than lung cancer, and only one additional study of associations between IAP and other cancers has been published since (Sapkota et al. 2008).

*Determine whether genetic susceptibility modifies associations between IAP and cancer.* Incorporating genetic analyses into intervention studies may improve risk assessment by clarifying whether the dose–response relationship between IAP and cancer differs for genetically susceptible subgroups. Toxicological and genetic studies also may identify or confirm chemical constituents or complex mixtures responsible for mediating the effects of IAP on cancer. For example, there is preliminary evidence of altered lung cancer susceptibility from indoor exposure to smoky coal associated with variants in glutathione *S*-transferase (*GST*) genes (Lan et al. 2000) and in genes involved in DNA repair (Shen et al. 2005). A meta-analysis (Hosgood et al. 2007) suggests that lung cancer risks associated with IAP exposure may be higher for persons with the *GSTM1*-null genotype, especially among populations in coal-using regions of China (Chan-Yeung et al. 2004; Lan et al. 2000). These studies have shown that coal emissions with the highest PAH levels have the strongest associations with lung cancer, a finding that is consistent with the PAH-type of mutation spectra found in the tumor protein p53 (*TP53*) and v-Ki-ras2 Kirsten rat sarcoma viral oncogene homolog (*KRAS*) genes of lung tumors from smoky-coal–exposed women (DeMarini et al. 2001).

Recent lung cancer genomewide association studies, which have been performed primarily in smoking Caucasian males, have reported associations between variants in loci *15q25*, *5p15*, and *6p21* and lung cancer susceptibility (Amos et al. 2008; McKay et al. 2008; Wang et al. 2008). The first genomewide association study of lung cancer among nonsmoking females in Asia reported that a variant in the *CLPTM1L-TERT* locus of chromosome 5 was strongly associated with lung cancer (Hsiung et al. 2010). The magnitude of the association was somewhat stronger than a previous estimate based on a genomewide association study of lung cancer among Caucasians, most of whom were smokers (Landi et al. 2009). Ongoing genomewide association studies of lung cancer in Asian populations should be able to provide new insights into how genetic susceptibility modifies the impact of IAP on lung cancer. Gene × environment interactions have been explored among populations exposed to smoky coal, and similar studies are needed in populations with exposures to IAP emissions from the combustion of other solid fuels including wood, dung, and crop residues.

*Determine whether effects of IAP are mediated via germline, somatic, and/or epigenetic changes.* This line of inquiry requires studies of early-life and life-course exposures, including studies of *in utero* exposures and studies to identify susceptible developmental stages. Rodent studies have shown that outdoor air pollution (Yauk et al. 2008) and cigarette smoke (Marchetti et al. 2011; Yauk et al. 2007) induce germ-cell mutations as well as epigenetic changes, and recent evidence also suggests that both these exposures are likely human germ-cell mutagens (DeMarini 2012; Somers 2011). Given that such exposures are not fundamentally different from many IAP exposures, effects of IAP exposures on germ-cell mutations and epigenetic changes in humans are plausible. If so, heritable risk resulting from household solid-fuel use could persist in populations even if exposures are reduced or eliminated. This possibility needs to be considered when evaluating intervention effectiveness, and reinforces the urgency of research in this area.

*Determine whether exposure to IAP via dermal and/or oral routes influence cancer risk.* IAP contaminates more than the air of closed structures; it may also settle onto the walls, floors, clothing, and food of the household inhabitants. A study conducted in Shanxi, China, confirmed the presence of genotoxic chemicals from indoor solid-fuel combustion in residential dust (Naufal et al. 2007). Transdermal exposures to PAHs are well documented from a variety of sources (Kammer et al. 2011). The majority of PAH exposure in western societies is from the diet (Ramesh et al. 2004), but there are no comparative studies of oral versus other routes of exposure to PAHs in IAP. Accounting for alternative routes of exposure in intervention studies will be necessary to ensure accurate results and for informing future interventions.

## Conclusions

IAP from indoor coal use increases the risk of lung cancer. Installing chimneys can reduce risk, and some genotypes, including *GSTM1*-null, may increase risk. Additional research is needed regarding the effects of IAP on cancers other than lung cancer and on the effects of using different types of solid fuels. We suggest incorporating the following specific research areas into intervention studies of IAP and cancer to maximize the information that can be obtained and improve risk assessment: studies of other cancers, studies of the influence of genotype and route of exposure, and studies to determine whether IAP induces heritable cancer risks via germline mutations or epigenetic changes.
